# Acute effects of cinacalcet on arterial stiffness and ventricular function in hemodialysis patients: A randomized double-blinded crossover study

**DOI:** 10.1097/MD.0000000000006912

**Published:** 2017-05-26

**Authors:** Aurélie Poulin, Pierre-Luc Bellemare, Catherine Fortier, Fabrice Mac-Way, Simon Desmeules, Karine Marquis, Valérie Gaudreault, Marcel Lebel, Mohsen Agharazii

**Affiliations:** a CHU de Québec Research Center, L’Hôtel-Dieu de Québec Hospital; b Department of Medicine, Faculty of Medicine, Université Laval, Québec, QC, Canada.

**Keywords:** aortic stiffness, brachial stiffness, calcium, carotid stiffness, chronic kidney disease, heart, hemodialysis, parathyroid hormone

## Abstract

**Background:**

Serum calcium concentration (Ca) plays an essential role in a vascular muscle tone and myocardial contractility. Previously, we showed that acutely lowering Ca by hemodialysis reduced arterial stiffness. Cinacalcet is a calcimimetic that lowers Ca and parathyroid hormone (PTH). The aim of the present study was to examine whether acute lowering of Ca by cinacalcet improves vascular stiffness and myocardial diastolic dysfunction.

**Method:**

This is a double-blinded randomized placebo-controlled crossover study that included 21 adult patients with end-stage kidney disease undergoing chronic hemodialysis. Subjects were assigned to placebo-cinacalcet (30 mg) or cinacalcet–placebo sequence. After each treatment period (7 days), aortic, brachial, and carotid stiffness were determined by examining carotid-femoral pulse wave velocity (cf-PWV), carotid-radial PWV (cr-PWV), and carotid distension. A central pulse wave profile was determined by radial artery tonometry and cardiac function was evaluated by echocardiography.

**Results:**

Cinacalcet reduced PTH (483 [337–748] to 201 [71–498] ng/L, *P* < .001) and ionized Ca (1.11 [1.08–1.15] to 1.05 [1.00–1.10] mmol/L, *P* = .04). Cinacalcet did not reduced cf-PWV significantly (12.2 [10.4–15.4] to 12.2 [11.0–14.6] m/s, *P* = .16). After adjustments for mean blood pressure, sequence, carryover, and treatment effects, cf-PWV was not significantly lowered by cinacalcet (–0.35 m/s, *P* = .139). There were no significant changes in central blood pressures, brachial and carotid stiffness, and echocardiographic parameters.

**Conclusion:**

In this study, 30 mg daily cinacalcet for 1 week did not have any significant impact on peripheral and central blood pressures, arterial stiffness parameters, or cardiac function (NCT01250405).

## 1 Introduction

Cardiovascular disease is the leading cause of death in hemodialysis patients, and arterial stiffness, as measured by carotid-femoral pulse wave velocity (cf-PWV), is now considered a new cardiovascular risk factor.^[1–3]^ There are 2 components to arterial stiffness: a structural stiffness and a functional stiffness. Structural arterial stiffness refers to the complexe alteration of the extracellular matrix by advanced glycation end-products, vascular hypertrophy, fibrosis, and calcification. Functional arterial stiffness refers to the changes of arterial stiffness that is explained by the pressure–diameter relationship or by the modification of vascular smooth muscle tone. Extracellular calcium concentration plays a key role in the contraction of vascular smooth muscle cells. We and others have previously shown that modulation of serum calcium, through changes in dialysate calcium concentration, has an impact on arterial stiffness parameters.^[4–10]^

Cinacalcet is a calcimimetic that reduces PTH and serum calcium concentrations, and it is used for the treatment of secondary hyperparathyroidism in CKD. Previous animal studies have suggested that the systemic activation of calcium sensing receptors by calcimimetics may produce acute effects on vascular tone and circulatory function.^[6,7]^ In addition, changes in the calcium concentration can significantly alter myocardial contractility, stroke volume, and cardiac output.^[11]^

We hypothesized that a reduction in serum calcium concentration by cinacalcet may result in a reduction of vascular smooth muscle tone and arterial stiffness. In the same fashion, a decrease in serum calcium concentration may reduce cardiac contractility, improving diastolic dysfunction. To test this hypothesis, we designed a placebo-controlled, double-blinded, crossover study, which examined the impact of cinacalcet on vascular stiffness and cardiac function.

## 2 Method

### 2.1 Study design and patient population

This is a randomized, double-blinded, placebo-controlled crossover study. The study was conducted at CHU de Québec Research Center between March 2012 and December 2013. Inclusion criteria were adult subjects on chronic (>3 months) hemodialysis with a dialysis vintage of < 3 years, PTH > 300 ng/L, corrected Ca > 2.10 mmol/L, stable antihypertensive drugs (> 1 month), stable doses of phosphate binders and dialysis calcium concentration, palpable femoral pulse, systolic BP of 90 to 180 mm Hg and expected survival of > 6 months. Patients were excluded if they had experienced an acute infection, myocardial infarction, or stroke within the past 3 months. They were also excluded if they were unable to provide informed consent, had intolerance to cinacalcet, or were not on the adequate birth control method. At a pre-randomization visit, blood samples were obtained for measurement of ionized calcium, phosphate, and PTH. The clinical and pharmacological data were recorded. Patients were randomized to their study sequence the following week. After each treatment period, arterial stiffness measures were performed along with echocardiography and assessment of mineral parameters.

### 2.2 Intervention, randomization, and masking

Cinacalcet (Sensipar) and the matching placebo were provided by Amgen. In the cinacalcet–placebo sequence, subjects received cinacalcet 30 mg/d for 7 days followed by placebo for 7 days. In the placebo–cinacalcet sequence, subjects received placebo for 7 days followed by cinacalcet 30 mg/d for 7 days. The duration of treatment was based on the terminal elimination half-life of 30 to 40 hours, and a steady-state concentration that is reached within 7 days.^[12]^ For subjects in the placebo–cinacalcet sequence, there was no reason for a washout period. In the cinacalcet–placebo sequence, 7 days (4.25 half-lives) were considered adequate to ensure that cinacalcet effects had faded. Simple randomization was performed by drawing the sequence (A or B) from a bag and was managed by the department of pharmacy.

### 2.3 Hemodynamic measurements

The tests were performed in a quiet room at a temperature of 22 to 24°C, between 8 AM and 11 AM, prior to their second dialysis of the week after a light breakfast without any coffee. The patients were in the supine position and allowed to rest for 15 minutes prior to the hemodynamic measurements. Brachial artery blood pressure (BP) was recorded using an automatic sphygmomanometer BPM-100 (BP-Tru, Coquitlam, Canada). BP was recorded 6 times, with a 2-minute interval between each measurement, and the average of the last 5 measurements was used for analysis. Radial artery tonometry was performed using the Sphygmocor system (AtCor Medical Pty. Ltd., Sydney, Australia). Systolic and diastolic brachial BP were used for calibration. Three consecutive recordings were performed. Central systolic BP (SBP), diastolic BP (DBP), mean BP (MBP), pulse pressure (PP) and the time of return of the reflected wave (Tr), pressure and time of first peak (P1 and T1) and second peak (P2 and T2), central augmented pressure (AP), and central augmentation index (AIx) (corrected for a heart rate of 75 beats per minute) were determined through the central pulse wave profile derived using the generalized transfer function.^[13]^ The mean central pulse wave profile for the entire group under each treatment is then constructed as a method to visually assess the modification of central pulse wave profile under each experimental treatment. This is performed by computing group mean DBP, pressure at the end of systole (ESP), pressure of the first and second pressure peak and their respective timing, Tr and duration of cardiac cycle in each treatment group.

#### 2.3.1 Pulse wave velocity

Aortic stiffness and brachial stiffness were determined by assessing carotid-femoral pulse wave velocity (cf-PWV) and carotid-radial pulse wave velocity (cr-PWV) using Complior SP (Artech Medical, Pantin, France). Direct measurements were used for assessing distance, and the maximal slope algorithm was used for determination of transit time. The average of 3 consecutive recordings PWV was used for the analysis.^[14]^

#### 2.3.2 Common carotid artery

The CCA systolic and diastolic diameters, intima-media thickness (IMT), and wall motion were measured by a high-resolution ultrasound (7.5-MHz transducer) using the echo-tracking system (ART.LAB, Esoate). Briefly, CCA was scanned for any stenosis or plaques, and measurements of CCA diameter and CCA IMT were performed in plaque-free arterial segments, 2 cm beneath the bifurcation over a distance of 1 to 1.5 cm. Three measurements were performed and averaged. The CCA-lumen cross-sectional area (LCSA) was calculated as LCSA = π (CCA diameter)^2^/4. The intima-media cross-sectional area (IMCSA) was calculated as IMCSA = π (CCA diameter/2+IMT)^2^ – π (CCA diameter/2)^2^, and wall/lumen ratio as (2 IMT/CCA diameter). CCA compliance, distensibility, and incremental elastic modulus (Einc) were determined from changes in CCA diameter during the systole and simultaneously measured CCA pulse pressure (PP) according to following formulas: CCA compliance = [π Dd(Ds – Dd)/2]/PP(m^2^ kPa^−1^ 10^–7^), CCA distensibility = 2[(Ds – Dd)/Dd]/PP(kPa^−1^ 10^–3^), and Einc [3(1+LCSA/IMCSA)]/CCA distensibility.^[15]^ Local carotid pulse pressure (PP) was determined by CCA tonometry after calibration using the diastolic and mean BP derived from radial artery tonometry as described above.

#### 2.3.3 Echocardiography

Echocardiography was performed in accordance to the American Society of Echocardiography recommendations for the use of echocardiography in clinical trials.^[16]^ Briefly, echocardiography was performed to determine left (LV) and right ventricular (RV) dimensions and function by using the following parameters: ejection fraction, stroke volume, end-systolic and end-diastolic volume of LV and RV, LV mass, DP/DT of LV and RV, tricuspid annular plane systolic excursion and excursion velocity, and Tei index of RV. To evaluate diastolic function of LV, the following parameters were studied: *E*/*A*, *E* wave on tissue Doppler, isovolumetric relaxation time, and deceleration time of *E* wave.

### 2.4 Study end-points

The primary end-point was to study the impact of cinacalcet treatment on mean BP adjusted cf-PWV. The secondary end-points were carotid-radial PWV, common carotid artery (CCA) stiffness, pulse wave profile, and cardiac function.

### 2.5 Laboratory

Ionised Ca was measured with an ion-selective electrode on a Nova PhoxPlus analyser. Intact PTH (1–84) was measured with the PTH stat assay from Roche diagnostics using 2 antibodies reactive with epitopes in the amino acid regions 26 to 32 and 37 to 42.

### 2.6 Sample size estimation

Based on our previous studies, a mean reduction of 0.1 mmol/L of ionized serum calcium was associated with a mean reduction of 0.8 m/s of cf-PWV. In clinical studies, 30 mg/d of cinacalcet reduced total calcium concentration by 0.2 mmol/L, corresponding roughly to 0.1 mmol/L of ionized serum calcium.^[17]^ Prior to the start of the study, sample size, based on a 2 × 2 crossover design, was calculated to detect a 0.8 mean difference (e.g., 13 ± 3.8 m/s vs 13.8 ± 3.8 m/s), between cinacalcet and placebo, using a correlation coeffecient of 0.96 (based on prior studies). Under the assumption of a significance level of 0.05 with a power of 0.80, and 2-sided hypothesis testing, a minimal sample size of 20 (10 patients in each sequence) was required. Assuming 3 dropouts for various reasons, we proposed that 23 subjects would provide sufficient power.

### 2.7 Statistical methods

Data are presented as median (25th–75th percentiles). Direct comparison of treatment effect was assessed by using the Wilcoxon signed-rank test where applicable. To evaluate whether there was a carryover effect and adjust cf-PWV for changes in mean blood pressure, we used a linear mixed model with fixed effects including periods (week 1 or 2), treatment (placebo or cinacalcet), and sequence (placebo–cinacalcet or cinacalcet–placebo), whereas a random effect for the patients nested in sequence was used. The dataset that was used for the analysis is available at https://doi.org/10.6084/m9.figshare.4794010.v1. SPSS 22 was used for data analysis. A 2-sided *P*-value of <.05 was considered to be statistically significant.

## 3 Results

### 3.1 Parameters of mineral metabolism

**Figure 1:**
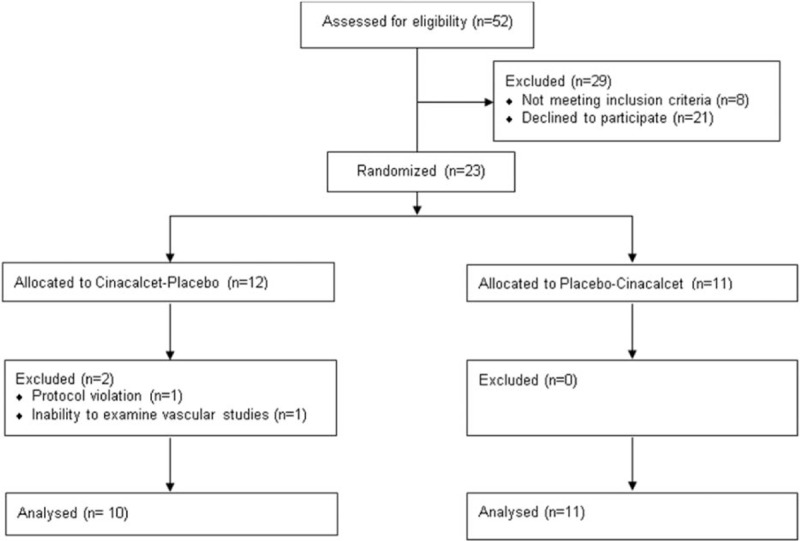
Study flow chart. The figure shows the study flow chart.

Figure 1 shows the study flow chart. Overall 21 subjects completed the study. The baseline characteristics of the subjects are presented in Table 1. After a 7-day administration of cinacalcet, corrected serum calcium decreased from 2.25 (2.16–2.37) to 2.13 (2.07–2.21) mmol/L (*P* < .001). Serum ionized calcium decreased from 1.11 (1.08–1.15) to 1.05 (1.00–1.10) mmol/L (*P* = .04) with a significant reduction in PTH levels as expected (Table 2).

**Table 1:**
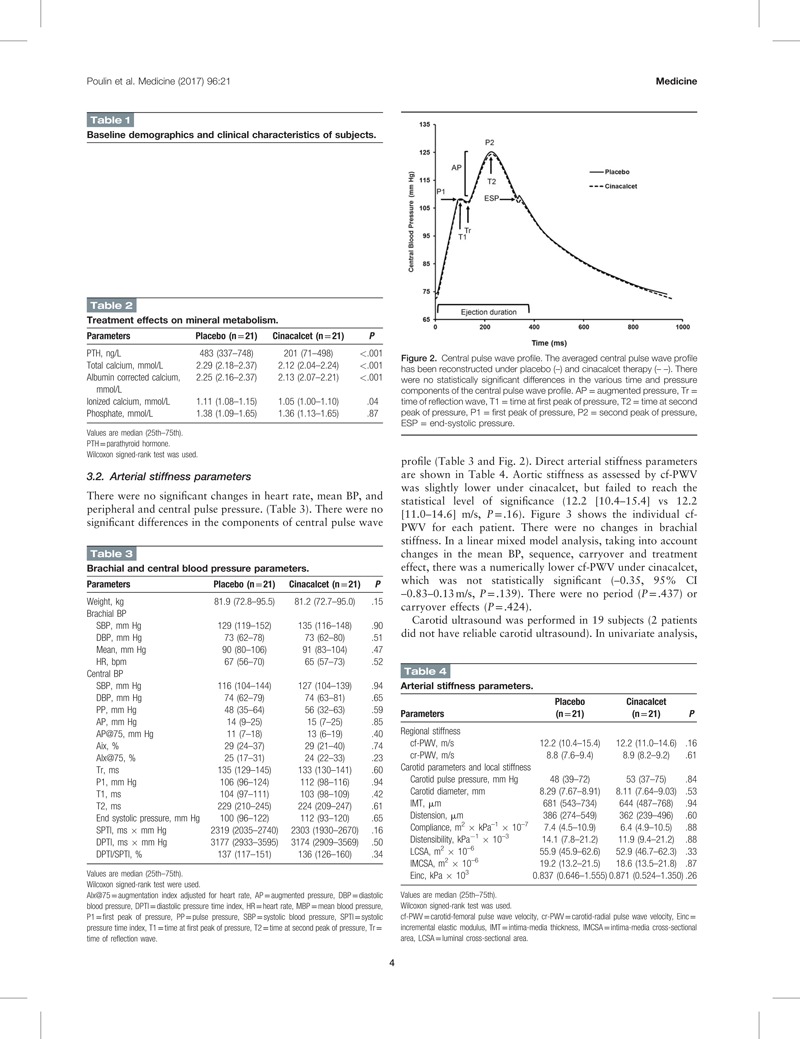
Baseline demographics and clinical characteristics of subjects.

**Table 2:**
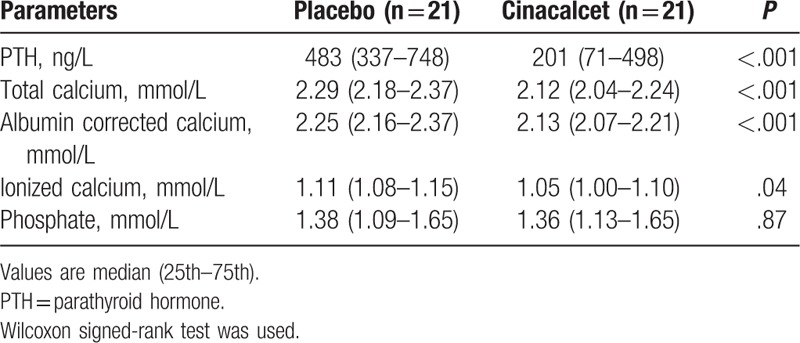
Treatment effects on mineral metabolism.

### 3.2 Arterial stiffness parameters

**Figure 2:**
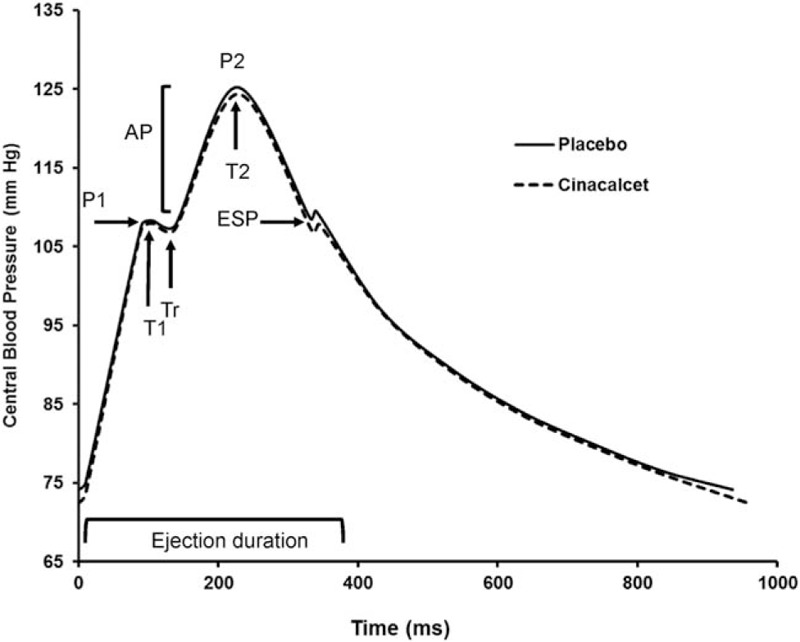
Central pulse wave profile. The averaged central pulse wave profile has been reconstructed under placebo (–) and cinacalcet therapy (– –). There were no statistically significant differences in the various time and pressure components of the central pulse wave profile. AP = augmented pressure, Tr = time of reflection wave, T1 = time at first peak of pressure, T2 = time at second peak of pressure, P1 = first peak of pressure, P2 = second peak of pressure, ESP = end-systolic pressure.

**Figure 3:**
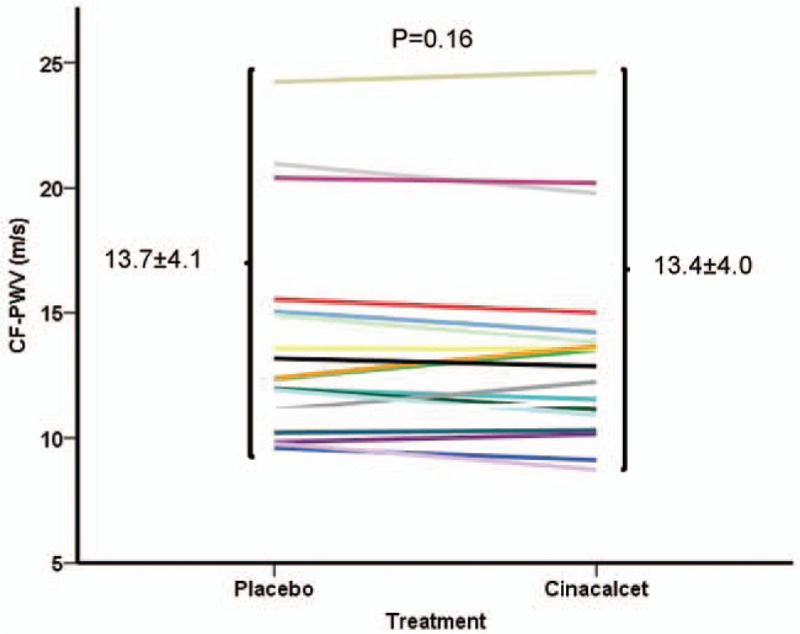
Aortic stiffness. Individual changes in aortic stiffness as measured by determination of carotid-femoral pulse wave velocity (cf-PWV) under placebo and cinacalcet. cf-PWV = carotid-femoral pulse wave velocity.

There were no significant changes in heart rate, mean BP, and peripheral and central pulse pressure. (Table 3). There were no significant differences in the components of central pulse wave profile (Table 3 and Fig. 2). Direct arterial stiffness parameters are shown in Table 4. Aortic stiffness as assessed by cf-PWV was slightly lower under cinacalcet, but failed to reach the statistical level of significance (12.2 [10.4–15.4] vs 12.2 [11.0–14.6] m/s, *P* = .16). Figure 3 shows the individual cf-PWV for each patient. There were no changes in brachial stiffness. In a linear mixed model analysis, taking into account changes in the mean BP, sequence, carryover and treatment effect, there was a numerically lower cf-PWV under cinacalcet, which was not statistically significant (–0.35, 95% CI –0.83–0.13 m/s, *P* = .139). There were no period (*P* = .437) or carryover effects (*P* = .424).

**Table 3:**
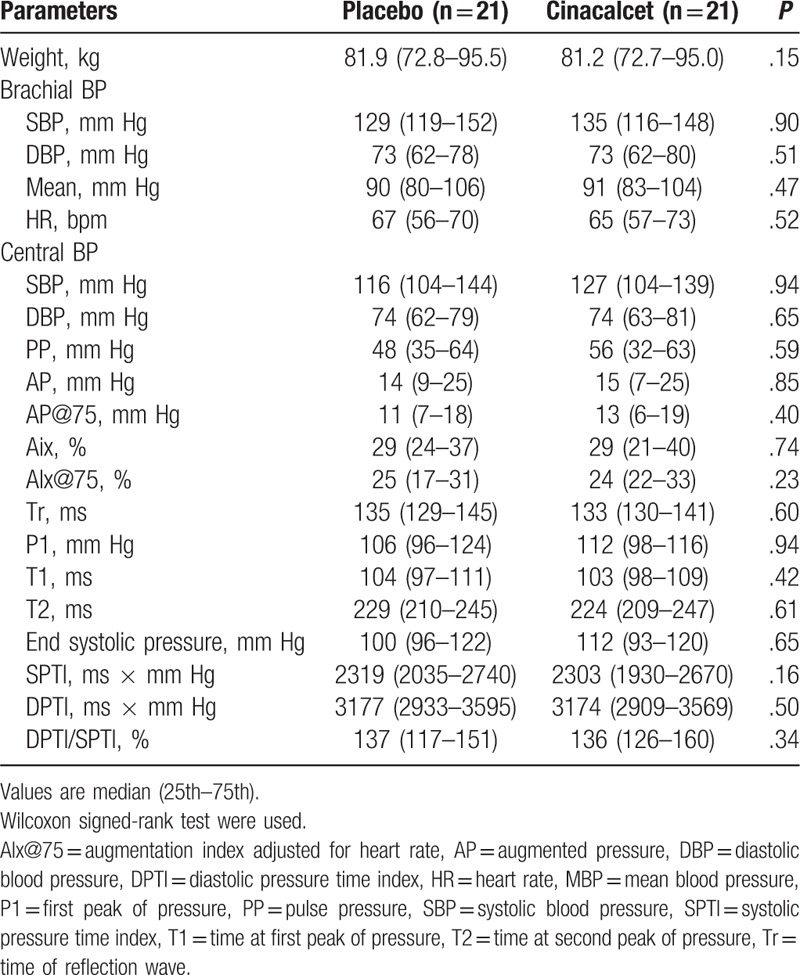
Brachial and central blood pressure parameters.

**Table 4:**
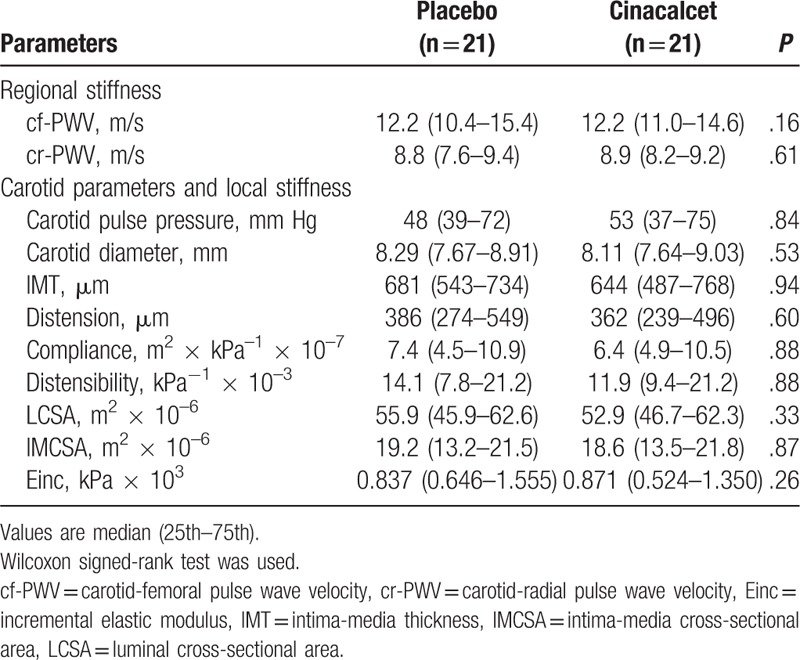
Arterial stiffness parameters.

Carotid ultrasound was performed in 19 subjects (2 patients did not have reliable carotid ultrasound). In univariate analysis, there were no statistically significant changes in the diameter, intima-media thickness, distension, compliance, and incremental elastic modulus. The results were not affected using the linear mixed model taking into account period, carryover, and treatment effects.

### 3.3 Cardiac parameters

Echocardiography was performed in 14 subjects on both visits (7 patients were unable to attend or were not echogenic enough to obtain reliable readings of the detailed parameters). In univariate analysis, there were no significant changes in cardiac output, stroke volume, and ejection fraction. However, left ventricular end-systolic volume (*P* = .64) and end-diastolic volumes were numerically higher but not statistically significant (*P* = .26). There were no significant changes in the parameters of diastolic dysfunction (Table 5). The results were not affected using the linear mixed model taking into account period, carryover, and treatment effects.

**Table 5:**
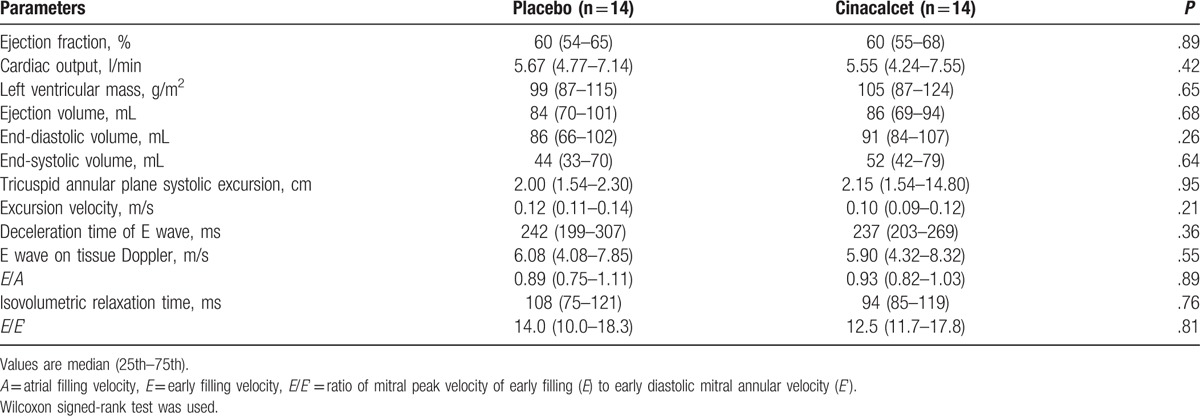
Cardiac parameters.

## 4 Discussion

To our knowledge, the present study is the first to investigate the acute effects of cinacalcet on detailed arterial stiffness parameters and ventricular function in hemodialysis patients using a randomized double-blinded crossover study. To examine our hypothesis, we chose a placebo-controlled crossover design for 2 reasons. First, it increased the power of the study as patients are their own controls. Second, such a design was essential in maintaining blindness of patients and study personnel. The results show, as expected, a reduction of PTH with the corresponding reduction of serum calcium in patients receiving cinacalcet 30 mg/d. However, we found no statistically significant changes in aortic, carotid, and brachial stiffness. In addition, we observed no statistically significant change in the cardiac function in the subset of patients that underwent echocardiography.

Although overall results are negative in this study, some limitations of the study need to be addressed. First, there was only a numerical reduction of cf-PWV by 0.35 m/s taking into account intra-individual changes in mean BP (*P* = .139). As stated in the methods, the sample size calculation in our study was based on the assumption that a reduction of 0.1 mmol/L in ionized calcium would result in a reduction of cf-PWV by 0.8 m/s. However, the reduction of ionized calcium level was smaller in our study (0.076 mmol/L). In reports where vascular stiffness was improved following acute variations in dialysate calcium concentrations, changes in the serum calcium concentrations were in general larger than those observed in the present study.^[9]^ For example, we had previously shown that dialysate calcium concentrations of 1.00, 1.25, and 1.50 mmol/L, changed serum ionized calcium concentrations by –0.14 ± 0.04, –0.02 ± 0.05, and 0.10 ± 0.06 mmol/L, respectively, and led to variations in post-dialysis vascular stiffness.^[9]^ Therefore, we may have missed a detectable change in vascular stiffness in the present study because of a more modest reduction in serum calcium concentration. Nevertheless, our results are in line with other studies in CKD that did not show any beneficial effect of chronic administration of cinacalcet on arterial stiffness parameters.^[18–20]^

Second, it is worth considering a potential confounding hemodynamic effect of PTH that is independent of serum calcium levels. As opposed to the reduction in serum calcium concentration by dialysis, which is accompanied by an immediate rise in PTH, the reduction of serum calcium concentration by administration of cinacalcet is accompanied by a reduction of PTH. The effects of PTH on the cardiovascular system are complex and still poorly understood, as PTH can directly act on vascular smooth muscle cells and cause vasorelaxation without changes in serum calcium concentration. Indeed, in animal models, the acute administration of PTH- or PTH-related peptide was shown to reduce BP and renal vascular resistance.^[21]^ In addition, selective vascular overexpression of PTH-related peptide in transgenic mice resulted in vasodilation and lower BP without a higher serum calcium concentration.^[22]^ Therefore, it remains unknown whether the lack of a statistically significant effect of cinacalcet on vascular stiffness is merely due to the small sample size or to a possible adverse vascular effect of a rapid reduction in PTH.

Third, cinacalcet, such as other calcimimetics, has been shown to have acute hypertensive and chronic hypotensive effects. In rat models, it was shown that acute administration of the calcimimetics cinacalcet and R-568 produce a sudden rise in BP. This effect may be mediated by a generalized vasoconstrictor response and may be dependent on both central and peripheral sites of action calcimimetics.^[6,7]^ However, in a recent analysis of the EVOLVE trial, investigators have shown that in an adjusted model, at the 20th week of the study, cinacalcet resulted in an additional reduction of systolic and diastolic BP of 2.2 and 1.3 mm Hg, respectively.^[23]^ In our study, however, we did not observe any impact of cinacalcet on any components of peripheral or central BPs.

Finally, calcium has been known to exert an inotropic effect on the myocardium. Lower levels of calcium can thus result in reduced myocardial contractility. However, the major potential benefit of cinacalcet in the EVOLVE trial was mainly driven by a reduction in sudden death and heart failure.^[24]^ In our study, the only change in the echocardiographic parameters was a slight increase in the left ventricular end-diastolic volume (86 [66–102] to 91 [84–107] mL) which was not statistically significant. Furthermore, the ventricular volume under cinacalcet was only available in a limited number of patients because of poor echogenicity and schedule conflicts, and are therefore exploratory in nature.

In conclusion, our findings do not support that 30 mg daily cinacalcet for 1 week has any significant impact on peripheral and central BPs, arterial stiffness parameters, and cardiac function in hemodialysis population.

## Acknowledgments

The authors are grateful to the dialysis personnel for their generous contribution and kind collaboration. They would like to thank Anne-Sophie Julien for her recommendations regarding statistical aspects.
